# Heteroatom Doped-Carbon Nanospheres as Anodes in Lithium Ion Batteries

**DOI:** 10.3390/ma9010035

**Published:** 2016-01-09

**Authors:** George S. Pappas, Stefania Ferrari, Xiaobin Huang, Rohit Bhagat, David M. Haddleton, Chaoying Wan

**Affiliations:** 1 Warwick Manufacturing Group, University of Warwick, Coventry CV4 7AL, UK; g.pappas@warwick.ac.uk (G.S.P.); r.bhagat@warwick.ac.uk (R.B.); 2 School of Aeronautics and Astronautics, Shanghai Jiao Tong University, Shanghai 200240, China; xbhuang@sjtu.edu.cn; 3 Department of Chemistry, University of Warwick, Coventry CV4 7AL, UK; d.m.haddleton@warwick.ac.uk

**Keywords:** carbon nanospheres, lithium batteries, organo phosphazene

## Abstract

Long cycle performance is a crucial requirement in energy storage devices. New formulations and/or improvement of “conventional” materials have been investigated in order to achieve this target. Here we explore the performance of a novel type of carbon nanospheres (CNSs) with three heteroatom co-doped (nitrogen, phosphorous and sulfur) and high specific surface area as anode materials for lithium ion batteries. The CNSs were obtained from carbonization of highly-crosslinked organo (phosphazene) nanospheres (OPZs) of 300 nm diameter. The OPZs were synthesized via a single and facile step of polycondensation reaction between hexachlorocyclotriphosphazene (HCCP) and 4,4′-sulphonyldiphenol (BPS). The X-ray Photoelectron Spectroscopy (XPS) analysis showed a high heteroatom-doping content in the structure of CNSs while the textural evaluation from the N_2_ sorption isotherms revealed the presence of micro- and mesopores and a high specific surface area of 875 m^2^/g. The CNSs anode showed remarkable stability and coulombic efficiency in a long charge–discharge cycling up to 1000 cycles at 1C rate, delivering about 130 mA·h·g^−1^. This study represents a step toward smart engineering of inexpensive materials with practical applications for energy devices.

## 1. Introduction

Scientific and industrial research in rechargeable batteries has been extended beyond the **“**conventional**”** lithium-ion batteries (LIBs) technology, driven by the ongoing development of high power demanding applications and tools [1]. Li-Air and Li-Sulfur (Li-S), having theoretical energy densities a few orders of magnitude larger than Li-ion commercial batteries, are the two major technologies predicted to move forward towards the realization of advanced batteries [1,2]. In the meantime, graphite-based LIBs still hold a prominent position since graphite is the only material to combine low cost, performance and easy processability—three factors which are crucial for industrial production and realization in true applications. The development and commercialization of advanced anode materials (silicon, transition metal oxides, various alloys, *etc.*) for Li-ion batteries is at the core of today’s efforts [3]. These materials have larger theoretical capacity than graphite (372 mA·h·g^−1^) but suffer mainly from low electrical conductivity and large volume expansion which lead to poor cycling performance.

Carbon (nano) spheres (C(N)Ss) have also been studied as active materials in various energy storage and conversion applications such as supercapacitors [4,5], hydrogen storage cells [6], catalysis [7,8] and lithium ion batteries [9,10]. In LIBs, Li-alloy-core-carbon-shell structures have been increasingly reported as composite anode materials, since the carbon layer can limit the issues of the alloy anodes mentioned above. Nevertheless, bare CSs (with or without heteroatom doping) have also attracted some interest in this field as an alternative anode to graphite. Wang *et al.*, prepared dense nitrogen-doped CNSs by carbonization of polypyrrole nanospheres. The CNSs, with diameters between 60 and 70 nm and specific area 59 m^2^·g^−1^, delivered a reversible capacity of 380 mA·h·g^−1^ at a current density of 60 mA·g^−1^ after 60 cycles and showed good high-rate performance delivering 200 mA·h·g^−1^ at 3 A·g^−1^ [11]. Xiao *et al.*, synthesized hydrogenated CNSs by a low temperature solvothermal method using CHCl_3_ as carbon source. Compared to Wang’s work, the obtained nanospheres had similar textural characteristics (particle size and specific area) and semi-graphitized structure but higher electrochemical performance (978 mA·h·g^−1^@50 mA·g^−1^ after 50 cycles) which was attributed to the high hydrogen-doping favoring the Li binding [12].

In order to increase the storage capacity and shorten the pathway length of Li ion diffusion, many groups have explored more sophisticated structures such as hierarchically porous [13,14] and hollow [15,16] carbon spheres. Recently, porous CNSs with single or double hollow architecture were prepared via a hard template method followed by carbonization and subsequent etching of the inorganic template. Compared to the single hollow nanospheres, the double shelled nanospheres showed improved cycling and rate performance ascribed to their unique hollow-in-hollow structure [17]. An unfavorable consequence of these engineered CSs (besides the multistep synthesis) is the high irreversible capacity loss at the first cycle. The increased reactivity due to the high surface area usually causes the fast decomposition of the electrolyte and a thick solid-electrolyte interface (SEI) layer formation [18]. Zhang *et al.*, prepared double shelled CNSs doped with nitrogen and showed their better electrochemical performance than the un-doped nanospheres when tested both in lithium ion and sodium ion cells [19]. It is obvious that not only the control over the textural structure but also the substitution of carbon by heteroatoms has a significantly positive effect in the performance of carbon materials in LIBs.

The synthetic methods for the formation of spherical particles, organic, inorganic or hybrid, relies on the chemical nature of the precursors and the desirable structure and properties of the final material which subsequently define the reaction conditions [20]. The one-step synthesis of highly cross-linked hybrid organo(cyclotriphosphazenes) (OPZs) is an excellent and highly efficient method to prepare various nanostructured materials such as nanospheres, nanotubes, core-shell and hollow particles. This method was first reported by Tang and Huang for the synthesis of OPZ nanotubes and microspheres and since then it has been extensively studied and applied to various materials applications [21,22]. Briefly, the OPZs formation proceeds through a polycondensation reaction between the hexachlorocyclotriphosphazene monomer and a co-monomer (cross-linker) with two -OH or -NH_2_ groups, in the presence of triethylamine (TEA). Control over the size and morphology can easily be achieved by varying the concentration and ratio of the monomers, the chemical structure of the co-monomer, the type of the solvent and sonication power [23,24]. The as-prepared OPZs have a hybrid organic–inorganic structure which, after carbonization under inert atmosphere, can provide heteroatom doped-carbons [25]. The ambient conditions, the fast rate of reaction and the low cost are the main advantages of this synthetic method.

Herein, we report the preparation of OPZs with diameters of approximately 300 nm and narrow dispersity by optimizing the reaction conditions. The equivalent heteroatom co-doped (nitrogen, phosphorous and sulfur) CNSs were obtained by carbonization of the as-prepared OPZs and their performance as anodes in a lithium half-cell is evaluated.

## 2. Results and Discussion

**Figure 1:**
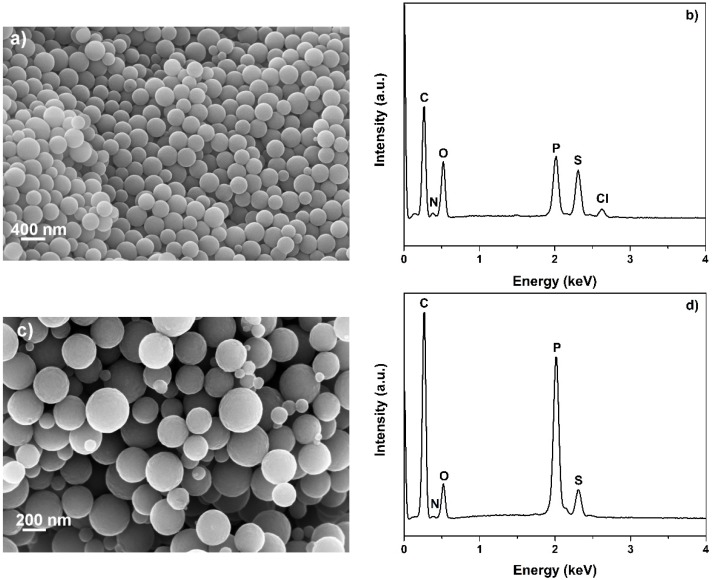
(**a**) SEM image of the organo (phosphazene) (OPZ) nanospheres (×50k magnification); (**b**) electron dispersive spectroscopy analysis (EDS) spectra of the OPZ nanospheres; (**c**) SEM image of the carbon nanospheres (CNSs) after carbonization at 850 **°**C (×100k magnification); (**d**) EDS spectra of the CNSs.

OPZs were successfully prepared by a single step polycondensation reaction of HCCP and BPS in acetonitrile under ultrasonication at ambient conditions. TEA was added to initiate the reaction which is characterized by a fast rate with completion within seconds. The HCCP/co-monomer ratio and HCCP concentration are two important factors controlling the size of the OPZ particles [26]. Here, a 3.5:1 BPS to HCCP molar ratio was selected while the HCCP concentration was 2.25 mg/mL. The resulting nanospheres have relatively narrow size dispersity with diameters ~330 nm and a relatively smooth surface (Figure 1a). The presence of some smaller nanospheres <170 nm is unavoidable but can be minimized by carefully positioning the flask in the sonicator in order that the sonication power is evenly distributed in the solution. The EDS (Electron Dispersive Spectroscopy) spectrum showed the presence of P, N, S, C, O and some remaining Cl originating from unreacted P-Cl sites of the HCCP ring (Figure 1b).

CNSs were obtained by carbonization of the as-prepared OPZs at 850 °C. The structure of the particles remained intact while an overall decrease in diameter is observed due to the weight loss and shrinkage during carbonization at high temperature (Figure 1c). From the EDS analysis (Figure 1d) it is evident that the derived carbon nanospheres are co-doped with N, P and S, showing the advantage of this method to produce carbons doped with multiple heteroatoms in one-pot synthesis. It is worth mentioning that the doping of carbon materials with multiple heteroatoms is desirable for energy storage applications since the presence of heteroatoms not only affects the textural characteristics but also alters the electronic properties of the carbon matrix [27]. The weight loss percentage between 120 and 850 °C calculated from the TGA curve is 56 wt % and two decomposition steps at T_onset_ = 478 °C and T_onset_ = 802 °C were observed (Figure S1). The first weight loss is attributed to the decomposition of the cross-linked HCCP/BPS structure and its initial conversion to an amorphous/low graphitized carbon structure. At higher temperature the transformation of amorphous to graphitic structure continued simultaneously with some heteroatom removal from the structure.

**Figure 2:**
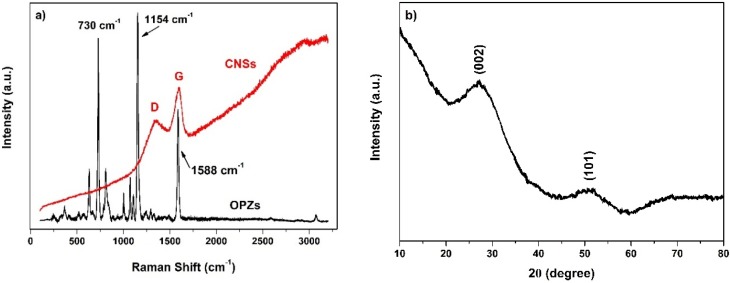
(**a**) Raman spectra of the OPZ and the CNSs; and (**b**) XRD pattern of the CNSs.

The chemical structure of the cross-linked OPZs was characterized by ATR-FTIR (Attenuated Total Reflectance Fourier Transform Infrared Spectroscopy) (Figure S2). The arrows indicate the major vibrational peaks attributed to the substituted phosphazene ring and the 4,4′-sulfonyl diphenol while, the peak at 935 cm^−1^ is assigned to the P-O-C_(aromatic)_ asymmetric stretch vibration indicating the successful reaction of the phenolic hydroxyls with the reactive P-Cl from the HCCP ring. Complementary to infrared spectra the Raman spectra of the OPZ nanospheres before and after carbonization was recorded (Figure 2a). The near infrared laser (785 nm) was selected to record the spectra of OPZs since the visible laser (532 nm) produced significant fluorescence and the vibration peaks were not visible. There are three main peaks at 730, 1154 and 1588 cm^−1^ attributed to C-S, O=S=O, and aromatic C-C stretching, respectively. After carbonization, the peaks arising from HCCP and BPS are absent and the two broad peaks are observed centered at 1597 cm^−1^ (G band) and 1347 cm^−1^ (D band), which belong to carbon *sp*^2^ and *sp*^3^ electronic configurations, respectively. The ratio of I_D_/I_G_ is 0.75 which is representative for pyrolized amorphous or partially graphitized carbons (hard carbons) and to some extent of defected turbostratic graphitic structure [28]. The results from Raman spectroscopy are further supported by the XRD pattern of the carbon nanospheres (Figure 2b). There are two broad peaks centered at 27 and 50° 2θ assigned to the (002) and (101) diffraction planes of hexagonal carbon layers (JCPDS, No. 75-1621). Similar to the Raman results, the broadening of the peaks is due to the disordered and highly defected structure of the material.

**Figure 3:**
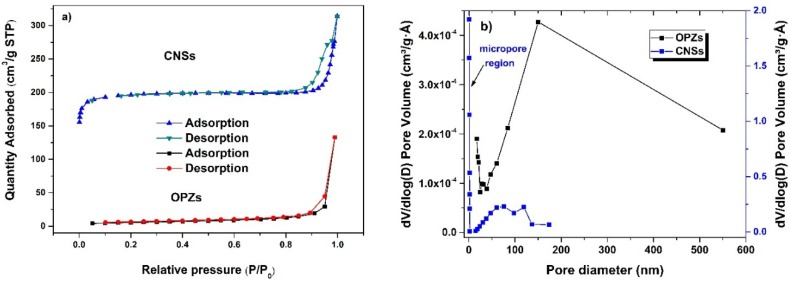
(**a**) N_2_ adsorption-desorption isotherms; and (**b**) pore size distribution of the OPZs and CNSs.

The N_2_ adsorption isotherm of the OPZ nanospheres shows a very slow increase in N_2_ adsorption up to 0.95 of the relative pressure (P/P_0_) where a steep increase of the adsorbed volume is observed and capillary condensation takes place (Figure 3a). This behavior is common in non-porous and/or macroporous materials (<50 nm diameter). Since no pores of this scale are observed in SEM analysis, the existence of macropores is originated from the textural void space between the nanospheres. This result is also evident from the Barrett-Joyner-Halenda (BJH) pore size distribution which is very broad up to relatively unlimited pore sizes (Figure 3b, black line). The specific surface area, calculated by the Brunauer-Emmett-Teller (BET) equation in the range 0.05–0.2 P/P_0_, is 19 m^2^·g^−1^. After carbonization, the CNSs showed a mixed type of isotherm curves. At low P/P_0_ < 0.05 a type-I isotherm with a high initial N_2_ uptake is observed which is characteristic of microporous materials. The adsorbed volume remains constant up to 0.9 P/P_0_ where the filling of large pores takes place (type-IV). The desorption-branch forms a H1-type hysteresis loop which closes just below 0.8 P/P_0_ indicating the presence of a wide range of mesopores. The specific surface area of CNSs is 875 m^2^·g^−1^ and was calculated by the Langmuir equation since the BET method resulted in a negative C constant value in the range 0.05–0.35 P/P_0_ which significantly demeans the true result. According to V-t plot calculations between 0.1 and 0.2 P/P_0_, the micropore area is 808 m^2^·g^−1^ and the major contributor of the total specific surface area. The pore size distribution of the CNSs shows a wide range of mesopores and an incomplete distribution in the micropore region (limitation of the BJH method). The high micropore surface area is a result of “defected” carbon structure due to the presence of heteroatoms. Additionally, the presence of the small quantity of Cl in the OPZs, could also affect the textural structure during carbonization. The large mesopores and macropores could be attributed to some aggregation between the CNSs during the carbonization. The total pore volume of CNSs is 0.43 cm^3^·g^−1^ and the micropore volume is 0.27 cm^3^·g^−1^.

**Figure 4:**
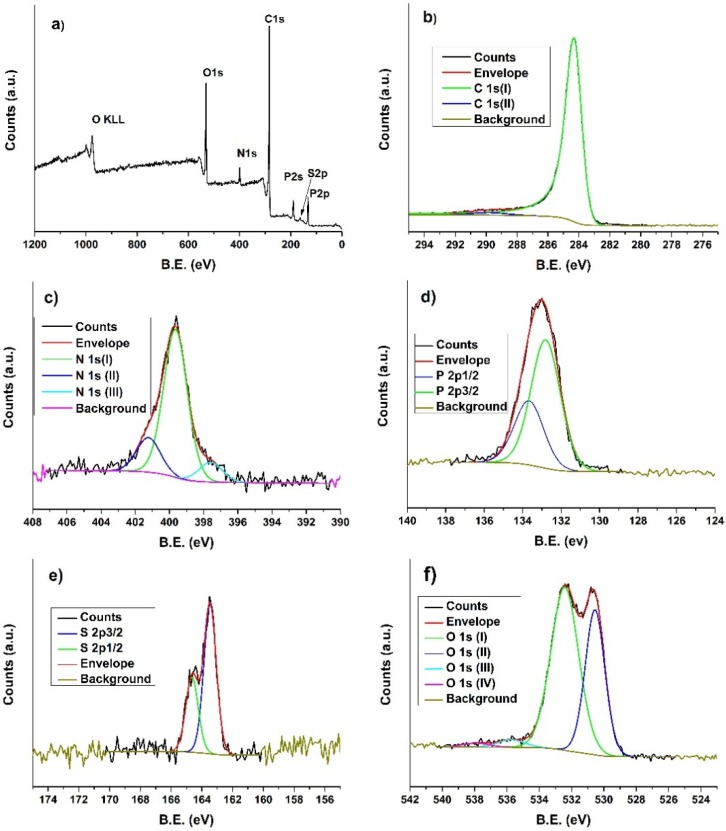
XPS of the CNSs sample: (**a**) survey spectrum and fitted high resolution spectra of; (**b**) C 1*s*; (**c**) N 1*s*; (**d**) P 2*p*; (**e**) S 2*p*; and (**f**) O 1*s*.

XPS analysis was performed on the CNSs in order to investigate the chemical composition and the relative concentration, and give information regarding chemical bonding configurations in the doped carbon nanospheres. The survey spectrum of the sample (Figure 4a) evidenced peaks of the expected doping elements, N, S, P together with C and O. This result is a clear indication of the successful incorporation of N, S and P atoms within the carbon spheres (in agreement with EDS analyses). The atomic percentage of the elements is reported in Table 1 together with the peaks position and width.

**Table 1:** Atomic percentage, peak position and width obtained by fitting the high resolution XPS spectra.

Element	at %	Peak Position (eV)	Width (eV)
C 1*s*	67.0	(I) 284.3	1.2
		(II) 289.8	2.8
O 1*s*	19.6	(I) 532.4	2.0
		(II) 530.5	1.4
		(III) 535.7	1.9
		(IV) 537.9	1.9
P 2*p*	8.0	(3/2) 132.8	1.8
		(1/2) 133.6	1.8
N 1*s*	4.7	(I) 397.6	1.6
		(II) 399.6	1.6
		(III) 401.2	1.6
S 2*p*	0.7	(3/2) 163.4	0.9
		(1/2) 164.6	0.9

It can be observed that—apart from O—among the other heteroatoms, P has the highest atomic percentage, while S is present in less than 1 at %. The high at % of P is related to: (i) the higher bonding degree of P in the initial OPZs’ structure; (ii) the lower “diffusion” ability through the carbon structure due to the larger atomic radius of P compared to N or S; and (iii) partial oxidation of P during carbonization. Peak fitting was performed using multiple components and the P 2*p* and S 2*p* curves were fitted taking into account the spin-orbit splitting and ratio 2*p*_1/2_:2*p*_3/2_ components of 0.5.

The high resolution spectra of C 1*s* (Figure 4b) shows the typical graphitic carbon asymmetric peak-shape centered at 284.3 eV due to sp^2^ bonding, with an associated shake-up feature at 289.8 eV originating from the pi to pi* transition [29]. Examination of the N 1*s* spectrum shown in Figure 4c revealed three different components that following previous interpretations [30] can be assigned to pyridinic (397.6 eV), pyrrolic (399.6 eV) and substitutional (401.2 eV) N. Phosphorous doped carbons have been reported in a very small number of studies [27,31] and for this reason clear evidence for the bonding of P even in graphitic carbon nanomaterials is missing [32]. The P 2*p* spectrum reported in Figure 4d shows the existence of a single P bonding environment, with the 2*p*_3/2_ component centered at 132.8 eV. According to some authors, this can be attributed to P-O bonds, while usually a P 2*p* signature between 131 and 129 eV is assigned to C-P species (substitutional P) [33]. Sulphur was also successfully incorporated in the CNSs as confirmed by XPS analysis; the S 2*p* spectrum (Figure 4e) shows the S 2*p*_3/2_ and S 2*p*_1/2_ peaks at 163.4 and 164.6 eV respectively, which are consistent with C-S-C bonds such as in thiophene-S [7,34]. SO_x_ groups, which usually show peaks at higher binding energy (about 168 eV), were not detected here [7,34]. The O 1*s* spectral envelope (Figure 4f) contains several different components pertaining to different oxygen-containing species. While the components at higher binding energies are ascribed to atmospheric contamination (H_2_O, C-OH, COOH, *etc.*), the component at 530.5 eV is likely due to NO/CO groups as determined previously for carbon nanoparticles [35].

### Electrochemical Behavior

**Figure 5:**
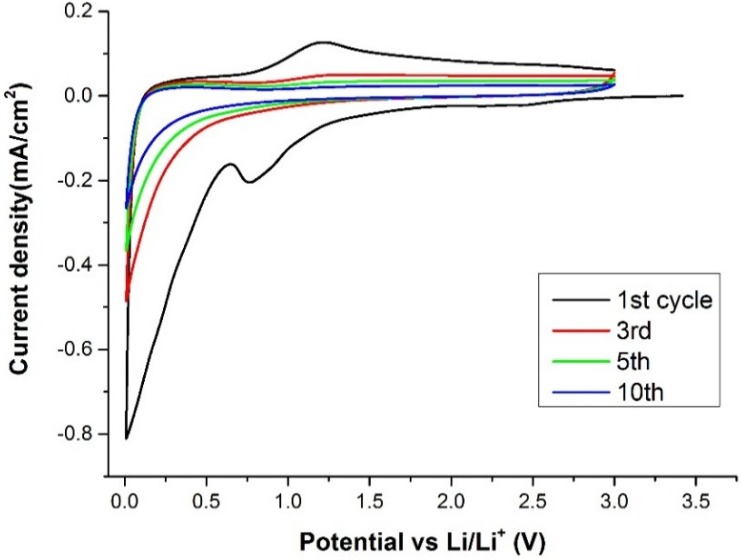
Cyclic voltammetry (CV) test of the CNSs. The graph shows some selected cycles between 5 mV and 3 V at a scan rate of 0.1 mV·s^−1^.

The lithium storage properties of the heteroatom-doped CNSs were evaluated in Swagelok and coin lithium half-cells by cyclic voltammetry and galvanostatic cycling for their potential application as anode material for lithium batteries. In Figure 5 the cyclic voltammetry (CV) test recorded at 0.1 mV·s^−1^ is reported. The first cycle shows a first cathodic peak at about 0.8 V that can be related to the formation of the SEI layer [16]. Also a second peak from about 0.2 V to 0.005 V is clearly observed, which can be assigned to the insertion of the Li ions in the pores of the nanospheres. A very broad anodic peak is also detected at about 1.0 V which could indicate that a reversible oxidation of some SEI components was taking place. For the subsequent cycles no other relevant reduction/oxidation peaks were observed. The voltammogram of this sample is in full agreement with previous reports in which SEI formation and Li insertion peaks were reported in the initial cycles [16,19].

**Figure 6:**
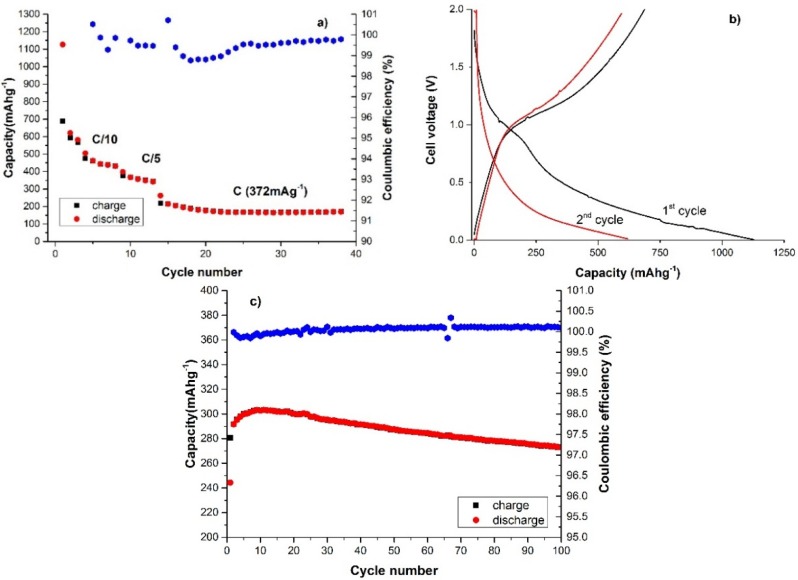
(**a**) Capacity *vs.* cycle number at C/20 (3 cycles), C/10 and C/5 (5 cycles each) and 1C current rate (25 cycles); (**b**) charge and discharge voltage profile of the first and second cycles at C/20; and (**c**) capacity *vs.* cycle number at C/5 current for 100 cycles.

The galvanostatic discharge/charge results are shown in Figure 6. The cells were cycled at different current rates up to 1C (Figure 6a) after three initial formation cycles at a current of C/20 (the theoretical capacity was considered 372 mA·h·g^−1^ as for a graphite electrode). A first discharge capacity of more than 1126 mA·h·g^−1^ (Figure 6b) was found with a large irreversible capacity of about 440 mA·h·g^−1^, as expected due to the large part of the initial discharge capacity related to the electrolyte decomposition and SEI formation on the anode. The high surface area of the CNSs can be considered responsible for the increased reactivity toward the electrolyte, which led to the observed irreversible capacity. In a previous work of Dahn’s group [36] the important role of the S and O atoms on the irreversible capacity of disordered carbons was evidenced. The authors found that the irreversible capacity loss increased by increasing the chalcogen content. In this work the S content is low and should have a minor effect on the capacity loss; the O content instead is significant and together with the high surface area is associated with the observed capacity loss after the first cycle. The charge and discharge voltage profile of the second and subsequent cycles are very similar with a steep voltage increase/decrease at higher current rates, which mainly suggests a pseudo-capacitive behaviour. This cell was then cycled for a further 100 cycles at the medium-low C/5 current rate (Figure 6c). After an initial recovery of the capacity that reached 300 mA·h·g^−1^ a continuous decline was observed with an overall 7% loss in discharge capacity for these cycles. However, a remarkable coulombic efficiency approaching 100% was obtained.

**Figure 7:**
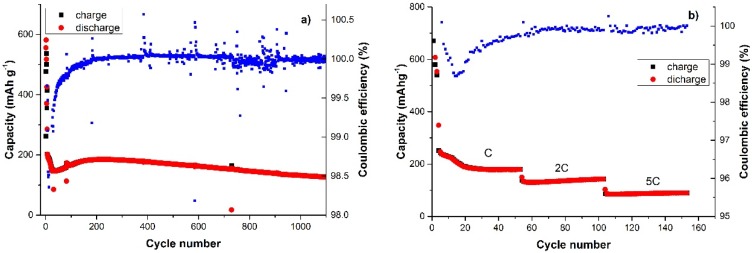
Capacity *vs.* cycle number at: (**a**) 1C rate for 1100 cycles; and (**b**) at 1C, 2C and 5C current rate for 50 cycles each.

The CNSs anode was then tested at 1C and higher C rates to check the rate capability. The cell was cycled at 1C for more than 1000 cycles (Figure 7a) and is well beyond the generally reported cycling for this type of materials for which no more than few hundreds of cycles are usually reported. The importance of this test has to be underlined since a novel anode material should be able to sustain a prolonged cycling for being considered competitive with the graphite anodes currently in use in commercial devices. The first few formation cycles confirmed the behaviour previously observed of an initial large irreversible capacity. A stable capacity value of about 180 mA·h·g^−1^ was reached and maintained for about 300 cycles, then a constant decay led to the final discharge value of 125 mA·h·g^−1^ for the last cycle.

The coulombic efficiency, although showing some fluctuation due to the experimental conditions, was definitely high with an average value of 99.992% (calculated over the whole cycling) that is the ideal efficiency required for a real application. In general, for all our tests, the efficiency was affected during the initial cycles by the formation of the SEI film and this is common for amorphous carbon also. The heteroatom-doped CNSs showed an efficiency among the highest reported in the literature so far indicating their excellent cycle stability. This good cycle performance could be achieved due to the unique porous structure of the CNSs which favoured strain relaxation during Li^+^ insertion/extraction and also to an appropriate surface area. At a higher C rate (5C) the discharge capacity was still around 90 mA·h·g^−1^ (Figure 7b). Although similar carbon systems have shown higher rate capabilities [16,19,37], the CNSs characterized herein for Li-ion cells, doped with N, P and S showed an interesting electrochemical performance and could represent a promising material concept. A synergetic effect between structure and heteroatom doping might be responsible for the observed high cycle stability. Heteroatoms are known to affect the electronic properties of carbon, the textural structure (porosity, disorder degree, crystal size) and also increase the active sites of the anode material [27]. All these effects are concomitant thus making it complicated to interpret the single contribution of all those factors to the electrochemical behavior of the CNSs. Although the nitrogen-doped carbons have been well studied in energy storage applications (see references herein), there is limited knowledge for other heteroatoms’ effect and even less for dual or ternary heteroatom doped carbons [27]. In this concept, further work is needed in order to clarify the doping effect on the electrochemical response optimizing the materials for real device applications.

## 3. Materials and Methods

### 3.1. Materials

Hexachlorocyclotriphosphazene (98%, Acros Organics, Pittsburgh, PA, USA), 4′-sulphonildiphenol (Fisher Scientific, Pittsburgh, PA, USA), triethylamine (Fisher Scientific) and acetonitrile (analytical grade, Fisher Scientific) were used as received. Acetone and deionized water were used for the washing steps.

### 3.2. Synthesis of Organophosphazene Nanospheres

The synthesis of the OPZs was performed according to literature [22,38] with a slightly modification in order to prepare particles of smaller diameter. Typically, a 250 mL round bottom flask containing 150 mL of acetonitrile, HCCP (2.25 mg/mL) and BPS (5.7 mg/mL), was placed in a sonicator bath (80W, 37 kHz) and the precursors left to dissolve before 4 mL of TEA were added. The particle formation was observed within 10 s and the mixture left under sonication for 10 min. The solids were collected by centrifugation, washed several times with acetone and water, and finally dried in vacuum oven at 50 °C for 24 h (yield 70%).

### 3.3. Carbonization

The conversion of the as-prepared OPZ nanospheres to carbon nanospheres took place in a tube furnace from 25 °C to 850 °C under N_2_ gas flow ~100 mLV·min^−1^ and a heating rate of 2.5 °CV·min^−1^. An isothermal step at 850 °C was maintained for 2 h and then the furnace left to cool down to room temperature.

### 3.4. Characterization

Field emission scanning electron microscopy (FE-SEM) and X-ray electron dispersive spectroscopy analysis (EDS) were performed on a Zeiss SIGMA SEM (Carl Zeiss AG, Oberkochen, Germany). Gold-Palladium (AuPd) sputtering was applied to the samples before observation. Carbonized samples did not need sputtering. FT-IR spectra were recorded on a Bruker Tensor 27 spectrometer equipped with an ATR cell (Bruker, Billerica, MA, USA). Raman spectra were recorded on a Renishaw in Via Confocal Raman Microscope equipped with 532 nm and 785 laser-lines, and a 0.75 NA (×50) lens was used (Renishaw, Wotton-under-Edge, UK). Thermogravimetric analysis curves were obtained with a Mettler Toledo instrument (Mettler-Toledo, Columbus, OH, USA) in the temperature range 25–850 °C at a heating rate of 10 °CV·min^−1^ under gas N_2_ flow. Specific surface area and pore size distribution were calculated from the nitrogen adsorption-desorption isotherm curves obtained with a Micromeritics ASAP 2020 Physisorption Analyzer (Micromeritics, Norcross, GA, USA). CNSs were degassed at 200 °C and OPZ at 60 °C for 16 h before measurements. X-ray Photoelctron spectroscopy (XPS) characterization was carried out with a Kratos Axis Ultra DLD spectrometer (Kratos Analytical Ltd, Manchester, UK) using monochromatic Al Kα source (*hν* = 1486.6 eV). Survey spectra were collected with a pass energy of 160 eV over a binding energy (BE) range of 1200–0 eV. High-resolution spectra were obtained using a 20 eV pass energy (resolution approximately 0.4 eV) and an analysis area of ~300 × 700 μm. The spectrometer was calibrated using the Fermi edge position of a polycrystalline Ag sample immediately prior to the experiments reported below. Peak fitting was performed by using CasaXPS software, using mixed Gaussian-Lorentzian (Voigt) line shapes and Shirley backgrounds.

### 3.5. Electrode Preparation and Electrochemical Characterization

To prepare the electrode, a slurry was made by mixing the carbon nanospheres with carbon black (Super P 65, TIMCAL, Cleveland, OH, USA) and poly(vinylidene fluoride) (PVDF, Solvay, Brussels, Belgium) in *N*-methyl-2-pyrrolidone (NMP, Sigma-Aldrich, St. Louis, MO, USA) with a weight ratio of 80:20:10. The obtained suspension was sonicated for one hour, then mixed on a magnetic stirrer for four hours and spread on a copper current collector by using a draw-down coater and a stainless steel applicator (bird applicator) with a 70 µm gap. The solvent was let to completely evaporate in a vacuum oven overnight and the foils were then transferred to a dry-room (humidity less than 1%, −45 °C dew point, Munters, Fort Myers, FL, USA). All cell components were dried in a vacuum oven (Binder Vacuum Drying Ovens with integrated vacuum pump system) at 50 °C overnight before assembly. The galvanostatic cycling were performed using 2032 coin cells from MTI (Richmond, CA 94804, USA); lithium metal was used as the counter electrode, and Celgard 2325 (Celgard, Charlotte, NC, USA) was used as the separator. To assemble the 2032 coin cells, the electrode foils were cut into disks of 1.2 cm diameter with a loading of about 3.5 mg/cm^2^ of active material. The electrolyte was 1 M LiPF6 in Ethylene Carbonate/ Ethyl methyl Carbonate (EC/EMC) 3:7 v/v and 1 wt % VC (PuriEl, Soulbrain MI, Northville, MI, USA). The cell were cycled at different current rate (from C/20 to 5C) in the 0.005–2 V range by using a Maccor Series 4000 battery cycler (Maccor, Tulsa, OK, USA). Swagelok cells with Li as reference electrode were also assembled for the cyclic voltammetry (CV) test. The CV was performed at a scan rate of 0.1 mV/s in the potential range 0.005–3.0 V using a Biologic VMP3 (Bio-Logic, Grenoble, France). The cells were tested at ambient temperature.

## 4. Conclusions

Heteroatom co-doped carbon nanospheres were successfully prepared by a carbonization process of highly cross-linked poly(cyclotriphosphazene-*co-*4,4′ sulphonyl diphenol) nanospheres. The co-doped carbon nanospheres have a mixed microporous/mesoporous structure and a high surface area of 875 m^2^/g. The XPS analysis showed a complex of heteroatoms in the carbon matrix and revealed the respective atomic concentration of S, N and P. The as-prepared CNSs showed remarkable cycling stability for more than 1000 cycles, delivering a capacity of about 130 mA·h·g^−1^ at a current rate of 1C. In addition, a remarkable coulombic efficiency as high as 99.99% was maintained during the long cycling thereby showing great promise for application in lithium batteries. The high specific surface area, the porous structure and multi-heteroatom doping all contributed to the electrochemical performance of the CNSs. Heteroatom doped CNSs are potential materials for various important applications in catalysis, supercapacitors and hydrogen storage and the synergistic effects of dopant atoms could be key to advanced technology.

## Supplementary Materials

The following are available online at www.mdpi.com/1996-1944/9/1/35/s1.
