# Supplementary material for: Heteroatom Doped-Carbon Nanospheres as Anodes in Lithium Ion Batteries
Source: Materials (Basel). 2016 Jan 9;9(1):35. doi: 10.3390/ma9010035 (PMC5456559; doi:10.3390/ma9010035)
Supplement: Supplementary file 1 [file materials-09-00035-s001.pdf]

Supplementary Materials: Heteroatom Doped-Carbon Nanospheres as Anodes in Lithium Ion Batteries

George S. Pappas, Stefania Ferrari, Xiaobin Huang, Rohit Bhagat, David M. Haddleton and Chaoying Wan

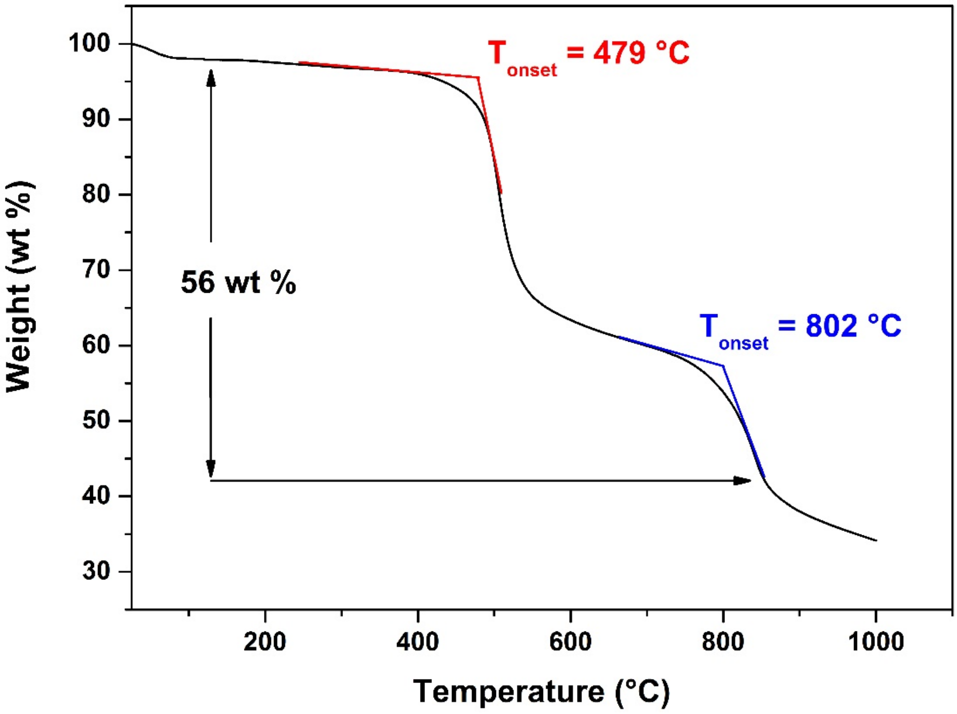

Figure S1. TG curve of the OPZs nanospheres.

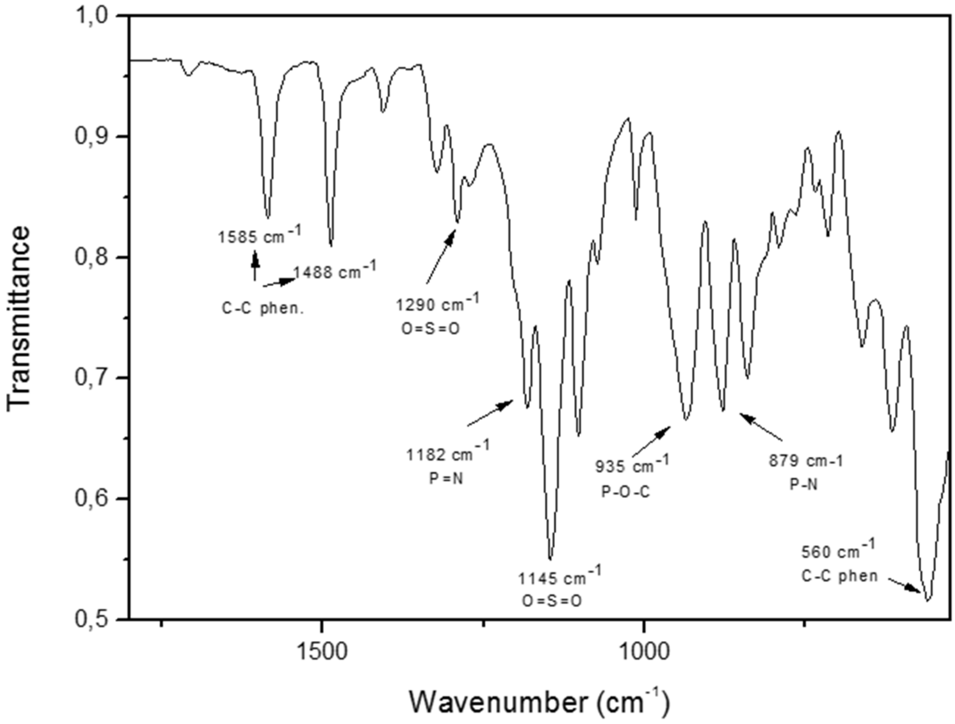

Figure S2. FT-IR Spectra of the OPZ nanospheres.
